# Pooled analysis of combination antiemetic therapy for chemotherapy-induced nausea and vomiting in patients with colorectal cancer treated with oxaliplatin-based chemotherapy of moderate emetic risk

**DOI:** 10.1186/s12885-021-08860-y

**Published:** 2021-10-16

**Authors:** Mototsugu Shimokawa, Toshinobu Hayashi, Junichi Nishimura, Taroh Satoh, Mutsumi Fukunaga, Reiko Matsui, Yasushi Tsuji, Fumitaka Mizuki, Takahiro Kogawa

**Affiliations:** 1 Department of Biostatistics, Yamaguchi University Graduate School of Medicine, 1-1-1 Minamikogushi, Ube, Yamaguchi, 755-8505 Japan; 2 Clinical Research Institute, National Hospital Organization Kyushu Cancer Center, Fukuoka, Japan; 3 Department of Pharmaceutical and Health Care Management, Faculty of Pharmaceutical Sciences, Fukuoka University, Fukuoka, Japan; 4 Department of Gastroenterological Surgery, Osaka International Cancer Institute, Osaka, Japan; 5 Department of Frontier Science for Cancer and Chemotherapy, Graduate School of Medicine, Osaka University, Osaka, Japan; 6 Department of surgery, Hyogo Prefectural Nishinomiya Hospital, Nishinomiya, Hyogo Japan; 7 Department of Pharmacy, National Cancer Center Hospital EastChiba, Kashiwa, Japan; 8 Department of Medical Oncology, Tonan Hospital, Sapporo, Hokkaido Japan; 9 Center For Clinical Research, Yamaguchi University Hospital, Yamaguchi, Japan; 10 Division of Early Clinical Development for Cancer, Advanced Medical Development Center, Cancer Institute Hospital of Japanese Foundation for Cancer Research, Tokyo, Japan

**Keywords:** Chemotherapy, Nausea, Vomiting, Colorectal cancer, Antiemetics, Oxaliplatin

## Abstract

**Background:**

Among patients with colorectal cancer (CRC) treated with oxaliplatin (L-OHP)-based chemotherapy, delayed chemotherapy-induced nausea and vomiting (CINV) have not been well controlled.

**Methods:**

We pooled data from two prospective observational studies in Japan and one phase III clinical trial to assess whether delayed CINV could be controlled with a combination of three antiemetics adding a neurokinin-1 receptor antagonist and identified individual risk factors, using an inverse probability treatment-weighted analysis.

**Results:**

A total of 661 patients were evaluable in this study (median age: 64 years; 391 male, and 270 female). 3 antiemetics controlled delayed nausea (33.18% vs. 42.25%; *p* = 0.0510) and vomiting (4.15% vs. 16.08%; *p* < 0.0001) better than with 2 antiemetics. Female and 2 antiemetics were risk factors for both delayed nausea (female—odds ratio [OR]: 1.918; 95% confidence interval [CI]: 1.292–2.848; *p* = 0.0012; 2 antiemetics—OR: 1.485; 95% CI: 1.000–2.204; *p* = 0.0498) and delayed vomiting (female—OR: 2.735; 95% CI: 1.410–5.304; *p* = 0.0029; 2 antiemetics—OR: 4.551; 95% CI: 2.116–9.785; *p* = 0.0001).

**Conclusions:**

Identifying individual risk factors can facilitate personalized treatments for delayed CINV. We recommend a 3-antiemetic combination prophylaxis for CRC patients treated with L-OHP-based chemotherapy, especially for female patients.

## Background

Colorectal cancer (CRC) is the third most commonly diagnosed cancer in the world [1]. Oxaliplatin (L-OHP)-based chemotherapy regimens, such as FOLFOX (5-fluorouracil + leucovorin + L-OHP) or XELOX (capecitabine + L-OHP) are preferred standard treatments for CRC [2–5]. Whereas chemotherapy-induced nausea and vomiting (CINV) were less prevalent for CRC patients on fluorouracil-based chemotherapy, they are a common adverse event for patients treated with L-OHP. In the MOSAIC trial, among patients on the FOLFOX and fluorouracil + leucovorin (FL) regimens, respectively, 73.7 and 61.1% had all-grade nausea (grades 3–4: 5.1 and 1.8%); and 47.2 and 24.0% had all-grade vomiting (grades 3–4: 5.8 and 1.4%) [6].

CINV impairs patients’ quality of life and often causes delay or refusal of curative chemotherapy among such patients [7]. Antiemetic treatment has been greatly improved by the development of second-generation 5-hydroxytryptamine-3 receptor antagonists (5HT3RAs; e.g., palonosetron) and neurokinin-1 receptor antagonists (NK1RAs). International guidelines for antiemetic therapy [8–10] include those published by the Japanese Society of Clinical Oncology in 2010 [11] and revised in 2015 [12]. However, control of delayed CINV is an unsolved issue [13, 14]. Especially, patients who receive receiving either high (HEC) or moderate (MEC) emetogenic chemotherapy have a high incidence of CINV. Although these guidelines consistently recommend antiemetic prophylaxis with 5HT3RAs, steroids and NK1RAs for patients on HEC, [8–10] whether adding a NK1RA to a 5HT3RA and dexamethasone is beneficial for patients on MEC is controversial. Taking a combination of two antiemetics (2antiemetics)—5HT3RAs and steroids—before receiving L-OHP resulted in a 90% complete response (CR) for control of nausea and vomiting during the 24 h after chemotherapy. However, CR for delayed CINV (i.e., after 24 h and up to a week) decreased to 54% if an additional antiemetic agent was not prescribed. This finding implies a need for routine antiemetic prophylaxis for delayed CINV following L-OHP-based chemotherapy [13].

Risk factors associated with CINV were reported to include younger age, female sex, a history of CINV, and low alcohol consumption [15–23]. However, these results were based on analyses of patients with various cancers, including many patients with breast cancer. Identifying risk factors for CINV in patients with CRC is important to providing them with appropriate care. Combinations of 3 antiemetics—2 antiemetics and NK1RA—for L-OHP-based chemotherapy were tested with a few studies; their clinical benefit is still under debate [24]. Our previous study indicated that 3 antiemetics treatment for CRC patients treated with L-OHP alleviated delayed vomiting without decreasing delayed nausea, [25] and XELOX caused a higher rate of nausea than FOLFOX. We considered these findings to reflect the study’s small sample size. We hypothesized that 3 antiemetics treatment is independent prophylaxis for delayed CINV, and XELOX is similar to FOLFOX regarding CINV.

We therefore investigated whether delayed CINV were controlled with 3 antiemetics treatment, as well as risk factors for delayed CINV in CRC patients treated with L-OHP-based chemotherapy, based on two prospective cohort studies [25, 26] and one randomized trial [27] in Japan.

## Methods

### Patients and methods

We analyzed pooled patient-level data from two multicenter, prospective observational studies. The individual study results were previously published or presented in a conference (study A, UMIN000005971 [25]; study B, no registry number available [26]; SENRI trial, UMIN000006456 [27]), and their designs are summarized in Table 1. Two prospective observational studies and one phase III clinical trial conducted among patients in Japan who were scheduled to receive MEC regimens; all were approved by institutional review boards or independent ethics committees at each site where they were performed. Written informed consent was obtained from all participating patients before any related study procedure was initiated.

**Table 1:** Study/Trial summary

Study or trial	Number of cancer types	Patients, *n*	CRC patients on L-OHP-based chemotherapy
Study A	9	2068	160
Study B	4	400	157
SENRI Trial	CRC only	413	344

### Data collection

For all three studies, patients were required to be at least 20 years of age, have solid tumors, and be chemotherapy-naïve. Any nausea or vomiting that occurred between 24 h and 6–7 days from the day of receiving their anticancer agents was defined as delayed. Eligible patients took two antiemetics of palonosetron or older 5HT3 receptor antagonists (RAs): azasetron, ramosetron and granisetron, and dexamethasone, all of which were administered within one hour before the scheduled L-OHP-based chemotherapy regimens. Aprepitant was optional in addition to two antiemetics.

### Statistical analysis

Patient demographics and delayed CINV incidences were summarized using descriptive statistics or contingency tables, and compared using Student’s *t*-test or chi-square test. We used an inverse probability treatment-weighted (IPTW) model derived from a logistic regression model to balance out observable characteristics among the administered antiemetics. Independent risk factors for delayed CINV were also evaluated using logistic regression analysis with a backward elimination method. Observed incidences of efficacy outcomes were compared between the 2 antiemetics and 3 antiemetics groups that included NK1RA, using Cochran–Mantel–Haenszel tests. *P* < 0.05 was considered significant, and were two-sided. All statistical analyses were performed using SAS 9.4 (SAS Institute, Cary, NC, USA).

## Results

### Unweighted and weighted patient characteristics

We included 661 patients in this analysis (Study A: *n* = 160 [24.2%], Study B: *n* = 157 [23.8%], SENRI trial: *n* = 344 [52.0%]; 2 antiemetics: *n* = 441, 3 antiemetics: *n* = 220). Baseline characteristics, including age, sex, motion sickness, drinking habit, L-OHP regimen and number of antiemetics, are shown in Table 2. Unweighted and weighted patient characteristics, stratified by 2 antiemetics versus 3 antiemetics, are listed in Table 3.

**Table 2:** Patients’ baseline characteristics

Characteristics	Study A (*N* = 160)	Study B (*N* = 157)	SENRI trial (*N* = 344)	Overall (*N* = 661)
	*n* (%)	*n* (%)	*n* (%)	*n* (%)
Age				
< 65 years	89 (55.6)	92 (58.6)	160 (46.5)	341 (51.6)
≥ 65 years	71 (44.4)	65 (41.4)	184 (53.5)	320 (48.4)
Sex				
Male	92 (57.5)	88 (56.1)	211 (61.3)	391 (59.2)
Female	68 (42.5)	69 (43.9)	133 (38.7)	270 (40.8)
Motion sickness				
No	143 (89.4)	135 (86.0)	282 (82.0)	560 (84.7)
Yes	17 (10.6)	22 (14.0)	56 (16.3)	95 (14.4)
Unknown	0 (0.0)	0 (0.0)	6 (1.7)	6 (0.9)
Drinking habit				
No	126 (78.8)	78 (49.7)	245 (71.2)	449 (67.9)
Yes	34 (21.3)	79 (50.3)	93 (27.0)	206 (31.2)
Unknown	0 (0.0)	0 (0.0)	6 (1.7)	6 (0.9)
Regimen				
FOLFOX	97 (60.6)	79 (50.3)	83 (24.1)	259 (39.2)
XELOX	63 (39.4)	78 (49.7)	261 (75.9)	402 (60.8)
Antiemetics				
2	114 (71.3)	157 (100.0)	170 (49.4)	441 (66.7)
3	46 (28.8)	0 (0.0)	174 (50.6)	220 (33.3)

**Table 3:** Unweighted and weighted baseline characteristics of patients with oxaliplatin-treated colorectal cancer, by number of antiemetic regimens

Characteristics	Unweighted, *n* (%)			Weighted, %		
	2 antiemetics	3 antiemetics	*p*-value	2 antiemetics	3 antiemetics	*p*-value
Total	153 (100)	29 (100)				
Age						
< 65 years	238 (54.0)	103 (46.8)	0.0830	46.26	47.00	0.8756
≥ 65 years	203 (46.0)	117 (53.2)		53.74	53.00	
Sex						
Male	265 (60.1)	126 (57.3)	0.4873	56.86	57.14	0.9518
Female	176 (39.9)	94 (42.7)		43.14	42.86	
Motion sickness						
No	378 (86.7)	182 (83.1)	0.2181	82.91	82.95	0.9905
Yes	58 (13.3)	37 (16.9)		17.09	17.05	
Unknown	5 (1.1)	1 (0.5)				
Drinking habit						
No	281 (64.2)	168 (77.4)	0.0006	77.97	77.42	0.8909
Yes	157 (35.8)	49 (22.6)		22.03	22.58	
Unknown	3 (0.7)	3 (1.4)				
Regimen						
FOLFOX	180 (40.8)	79 (35.9)	0.2233	35.56	36.41	0.8541
XELOX	261 (59.2)	141 (64.1)		64.44	63.59	

Percentages of patients aged ≥65 years (unweighted) were 2 antiemetics: 46.0% and 3 antiemetics: 53.2% years. The 2 antiemetics group also included more patients with drinking habits (*p* < 0.0006) than the 3 antiemetics group.

Propensity scores between antiemetics groups were adequately balanced after IPTW adjustment, as patient characteristics of age (*p* = 0.8756), sex (*p* = 0.9518), motion sickness (*p* = 0.9905), drinking habits (*p* = 0.8909), and L-OHP regimens (*p* = 0.8541) were similar between the 2 antiemetics and 3 antiemetics groups.

### Control of CINV

**Fig. 1:**
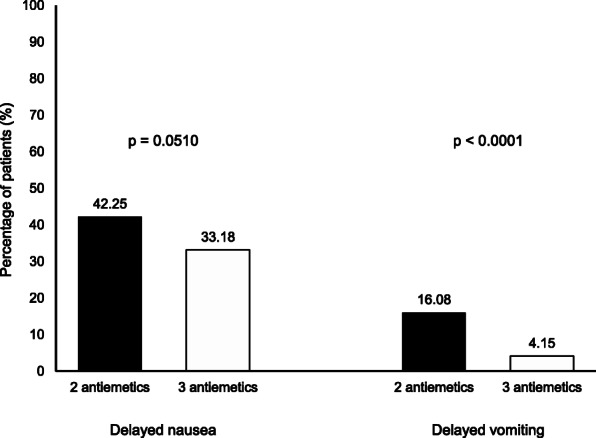
Incidence of delayed CINV. Incidences of delayed CINV were adjusted using IPTW method. Black bars show the 2 antiemetics group, while white bars indicate the 3 antiemetics group. The incidence of delayed vomiting was significantly lower in the 3 antiemetics group than that in the 2 antiemetics group (*p* < 0.0001)

The IPTW-adjusted CINV incidence is shown in Fig. 1. Although the cohort as a whole had a high incidence of delayed nausea (37.72%), the 3 antiemetics group had less delayed nausea (33.18%) than the 2 antiemetics group (42.25%), with borderline significance (*p* = 0.0510). Overall delayed vomiting incidence was low (10.13%), but significantly lower in the 3 antiemetics group (4.15%) than in the 2 antiemetics group (16.08%; *p* < 0.0001).

### Risk factors for CINV

We performed univariate and multivariate logistic regression analyses of risk factors for delayed CINV, including age, sex, motion sickness, drinking habit, L-OHP-based regimen and antiemetic regimen (Table 4).

**Table 4:** Risk factors for delayed CINV

	Delayed nausea				Delayed vomiting			
	Univariate		Multivariate		Univariate		Multivariate	
	OR (95%CI)	*p*-value	OR (95%CI)	*p*-value	OR (95%CI)	*p-*value	OR (95%CI)	*p*-value
Age: < 65 vs. ≥ 65 years	1.678 (0.903–3.116)	0.1014			1.976 (0.614–6.354)	0.2533		
Sex: female vs. male	1.909 (1.288–2.829)	0.0013	1.918 (1.292–2.848)	0.0012	2.636 (1.378–5.044)	0.0034	2.735 (1.410–5.304)	0.0029
Motion sickness: yes vs. no	1.921 (1.161–3.180)	0.0111			2.027 (0.993–4.138)	0.0524		
Drinking habit: yes vs. no	1.361 (0.843–2.196)	0.2069			1.712 (0.724–4.051)	0.2208		
Regimen: FOLFOX vs. XELOX	1.067 (0.713–1.597)	0.7529			1.616 (0.863–3.028)	0.1339		
2 antiemetics vs. 3 antiemetics	1.474 (0.998–2.176)	0.0514	1.485 (1.000–2.204)	0.0498	4.429 (2.074–9.460)	0.0001	4.551 (2.116–9.785)	0.0001

Known risk factors, [17–25] i.e., female sex, history of motion sickness and morning sickness, were identified as risk factors for delayed CINV, whereas patients who drank alcohol five times a week and who were older experienced CINV less frequently.

Female sex and use of only 2 antiemetics were associated with greater risks for both delayed nausea (female sex—OR: 1.918; 95% CI: 1.292–2.848, *p* = 0.0012; 2 antiemetics—OR: 1.485; 95% CI: 1.000–2.204, *p* = 0.0498) and delayed vomiting (female sex—OR: 2.735; 95% CI: 1.410–5.304, *p* = 0.0029; 2 antiemetics: OR: 4.551; 95% CI: 2.116–9.785, *p* = 0.0001).

**Fig. 2:**
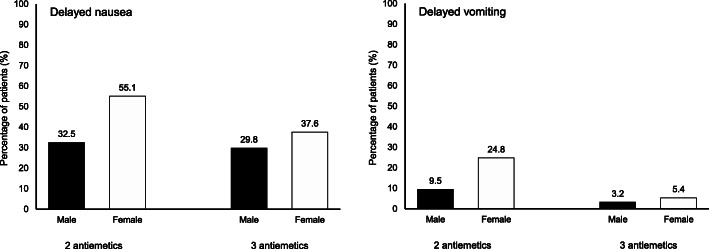
Incidence of delayed CINV by risk factor. The graph displays analyses of incidences of delayed CINV of male (black bars) and female (white bars) between the 2 antiemetics group and the 3 antiemetics group

Logistic regression analysis showed female sex and 2 antiemetics regimens to be common risk factors for delayed CINV. Women were more susceptible to CINV than men in every aspect investigated (Fig. 2).

## Discussion

The present study provides incidences of, and risk factors for, delayed CINV in CRC patients receiving L-OHP-based chemotherapy, based on three prospective studies [25–27]. Delayed nausea occurred frequently but was less common in patients who received three antiemetics adding an NK1RA than those who received two antiemetics. Delayed vomiting incidence was relatively low, and significant lower in the 3 antiemetics group. Multivariate analysis identified female sex and 2 antiemetics regimens as independent risk factors for delayed CINV.

Although adding a NK1RA to a 5HT3RAs and a steroid is well established and clearly recommended by all international guidelines for patients undergoing HEC, [8–10, 12] its benefit is still controversial for MEC other than carboplatin (CBDCA) -based regimens, and recommendations for using NK1RAs with MEC vary considerably among guidelines. As few clinical trials have attempted to clarify optimal antiemetic prophylaxis for CRC patients who receive L-OHP-based chemotherapy, evidence-based guidance in this setting is lacking.

Risk/benefit profiles and medication costs are important factors in treatment decisions, including antiemetic treatment. Choosing Wisely, an initiative of the American Board of Internal Medicine (ABIM) Foundation that seeks to advance a national dialogue on avoiding unnecessary medical tests, treatments and procedures, suggests that patients receiving MEC not use NK1RAs, for tolerability and economic reasons [28].

Iihara et al. [29] reported that use of two antiemetics—5HT3RA and dexamethasone—was sufficient for prevention of CINV in most MEC regimens, and found no significant differences in control of CINV among L-OHP, carboplatin and irinotecan. A recent meta-analysis [24] indicates that adding NK1RAs for patients undergoing L-OHP-based chemotherapy did not have a very pronounced effect. However, the two major studies that included patients with CRC with similar L-OHP doses showed conflicting results. In addition, one of them used casopitant, which was never approved due to safety concerns. Therefore, the results have to be interpreted with caution. Hesketh et al. [13] advocated the need for routine antiemetic prophylaxis for delayed CINV following L-OHP-based chemotherapy. Tsuji et al. [25] reported that delayed nausea incidence was still high for MEC, and patients on L-OHP-based regimens seemed to benefit from doublet therapy with palonosetron or triplet therapy with aprepitant. Nishimura et al. [27] reported that three antiemetics that included aprepitant was more effective than two antiemetics in preventing CINV in CRC patients on L-OHP-based regimens. In addition, the antiemetic effects of aprepitant did not significantly differ whether combined with palonosetron or not. In the present study, although the 2 antiemetics group included patients who received palonosetron, it was less effective than the 3 antiemetics regimens.

Within the MEC classification, L-OHP has relatively high risks of CINV, as well as CBDCA, which suggests a different antiemetic prophylaxis strategy is appropriate. In the last update of the MASCC/ESMO guidelines, [9] experts discussed different recommendations for CBDCA-based and L-OHP-based chemotherapies, as L-OHP-based chemotherapy is estimated to carry a high emetic risk within MEC. The MASCC/ESMO guideline indicates that a 10% difference in CINV rates would be noticeable to the patient, [30] and appears to be a reasonable threshold to warrant a change in clinical practice. In the present study, 3 antiemetics regimens reduced delayed vomiting incidence by 11.93% and delayed nausea incidence by 9.07%, compared with 2 antiemetics.

Younger age, female sex, a history of CINV, and low alcohol consumption have been reported as well-known risk factors [15–23]. Roscoe et al. [17] reported that a chemotherapy history was a stronger predictor than other predictors, including morning sickness, age, and motion sickness. Study cohorts for these reports included large percentages of breast cancer patients, whereas few studies of risk factors for CINV in CRC patients have been performed. Takemoto et al. [31] reported that female sex and aprepitant use were risk factors for CINV in CRC patients who received L-OHP-based chemotherapy, and that 3 antiemetics regimens that included aprepitant were more effective for women than for men in preventing CINV in this setting. Our integrated analysis showed that female sex and 2 antiemetics regimens were independent risk factors for both delayed nausea and delayed vomiting in CRC patients on L-OHP-based chemotherapy.

In the analysis focused on the efficacy of 3 antiemetics compared with 2 antiemetics containing palonosetron, a second-generation 5-HT3RA which is more effective than first-generation 5-HT3RAs against delayed CINV, 3 antiemetics was superior than 2 antiemetics for preventing delayed CINV, and female sex was identified as an independent risk factor for delayed vomiting [32].

In addition, the randomized trial in Chinese female patients with gastrointestinal cancer at high risk for CINV (younger than 50 years, no or low alcohol consumption) demonstrated significantly better antiemetic effect of 3 antiemetics, palonosetron plus dexamethasone plus aprepitant, compared with palonosetron plus dexamethasone for CINV caused by L-OHP or irinotecan-based chemotherapy [33].

On the other hand, the incremental benefits by adding aprepitant in men (3% delayed nausea, 6% delayed vomiting) was very small compared to women where the absolute benefits in delayed CINV are large in this study. These data suggest that 2 antiemetics for prevention of delayed CINV may be sufficient for men receiving L-OHP-based regimen. Although there may be some operational issues such as the complexity electronic order sets at medical institutions, it is worth considering individualizing antiemetic prophylaxis by gender.

Our study also found no significant difference between XELOX and FOLFOX regimens with respect to delayed CINV, as we hypothesized. Many physicians prefer XELOX as it does not need continuous 5-FU infusion, and our results support XELOX administration.

### Study limitation

The present study had some limitations. First, as its design was neither randomized nor blind, present findings should be interpreted within the limitations of the observational study design. Second, the two integrated studies had a bias in the number of patients, which we attempted to mitigate by using the IPTW method to balance the observable characteristics of the two antiemetic treatments. Despite these limitations, the findings describe CINV incidence and its risk factors in routine clinical practice, rather than in a controlled trial.

## Conclusions

This study clarified that female sex and use of only two antiemetics are risk factors of delayed CINV for CRC patients who undergo L-OHP-based chemotherapy. We recommend combining three antiemetics as prophylaxis for CRC patients treated with L-OHP-based chemotherapy, especially female patients.

## Data Availability

The data that support the findings of this study are available from two prospective observational studies and one phase III clinical trial but restrictions apply to the availability of these data, which were used under license for the current study, and therefore, the data are not publicly available. However, data are available from the authors upon reasonable request and with permission of all study groups.

## Abbreviations

CRC: Colorectal cancer
L-OHP: Oxaliplatin
CINV: Chemotherapy-induced nausea and vomiting
IPTW: Inverse probability treatment-weighted
OR: Odds ratio
CI: Confidence interval
FOLFOX: 5-fluorouracil + leucovorin + oxaliplatin
XELOX: Capecitabine + oxaliplatin
FL: Fluorouracil + leucovorin
5HT3RAs: 5-hydroxytryptamine-3 receptor antagonists
NK1RAs: Neurokinin-1 receptor antagonists
HEC: High emetogenic chemotherapy
MEC: Moderate emetogenic chemotherapy
CR: Complete response
RAs: Receptor antagonists
ABIM: The American Board of Internal Medicine
CBDCA: Carboplatin
MASCC/ESMO: Multinational Association for Supportive Care in Cancer/ European Society for Medical Oncology
